# Idiopathic Orbital Inflammation Treated With Rituximab Monotherapy

**DOI:** 10.7759/cureus.33614

**Published:** 2023-01-10

**Authors:** Amani Bawazeer, Wejdan Rahali, Alhanouf Alsharif, Malak Alshehri, Lama Maksood, Ahad Babkier, Walaa Hommadi, Basant Othman, Mohammed A Omair, Waleed A Hafiz

**Affiliations:** 1 Department of Medicine, Alnoor Specialist Hospital, Makkah, SAU; 2 College of Medicine, Umm Al-Qura University, Makkah, SAU; 3 Department of Neurosurgery, King Abdullah Medical Complex, Jeddah, SAU; 4 Department of Ophthalmology, Alnoor Specialist Hospital, Makkah, SAU; 5 Rheumatology Unit, Department of Medicine, King Saud University, Riyadh, SAU

**Keywords:** rheumatology, ophthalmology, rituximab therapy, occular diseases, idiopathic orbital inflammation

## Abstract

Idiopathic orbital inflammation is a benign condition that affects the orbit and the extra-orbital structures. It presents as ocular pain, peri-orbital swelling, proptosis, and restricted ocular movements. This condition is diagnosed based on clinical features that are then confirmed by radiological and histopathological findings. Corticosteroids are the initial therapy of choice. Disease-modifying antirheumatic drugs are added in steroid non-responsive patients. Rituximab is now becoming increasingly used as a third-line therapy for this disease. We report a case of idiopathic orbital inflammation treated with rituximab monotherapy without the use of corticosteroids or disease-modifying antirheumatic drugs.

## Introduction

Idiopathic orbital inflammation (IOI), also named orbital pseudotumor, is a benign inflammatory condition that is commonly limited to the orbit but can also extend to the extra orbital area [1,2]. It is a heterogeneous group of conditions characterized by significant orbital inflammation without obvious local or systemic etiology [2]. It can occur acutely, sub-acutely or chronically.

According to the anatomical location and the structures involved, IOI is classified into anterior, diffuse, posterior or apical, myositis, and dacryoadenitis. Periscleritis, perineuritis, and focal mass are other uncommon IOI subtypes [1].

Patients with IOI present with a broad range of clinical symptoms. The most prevalent presenting symptoms are pain and periorbital edema. Restricted ocular movement and conjunctival chemosis are other prevalent characteristics. Rarely, IOI is associated with other ocular conditions such as uveitis, exudative retinal detachment, papillitis, and proptosis [1].

IOI is a diagnosis of exclusion and is an uncommon clinical condition [2]. Detailed history, clinical examination, computerized tomography (CT), and magnetic resonance imaging (MRI) findings are combined to establish the diagnosis. Orbital biopsy is performed for accessible ocular lesions like dacryoadenitis [1]. For assessing the extent of the condition, contrast-enhanced MRI is commonly the most used modality of choice [3].

Management of IOI is mostly nonsurgical. While IOI is often steroid responsive, some patients' symptoms and signs are self-limited. Others require disease-modifying antirheumatic medications (DMARDs) as well as radiation [2,3]. Failure to respond to medical therapy and radiation necessitates surgical resection [3].

Rituximab (RTX) is a chimeric monoclonal antibody that binds to CD20 antigen and leads to B-cell inhibition. It has numerous therapeutic indications, with rheumatoid arthritis, small vessel vasculitis, non-Hodgkin lymphoma and leukemia being the most common ones [4].

In patients with inflammatory eye conditions including IOI following corticosteroid therapy, multiple DMARDs have been shown to improve these conditions. These include conventional synthetic DMARDs such as methotrexate, azathioprine, mycophenolate, and cyclophosphamide. Biological DMARDs such as adalimumab and RTX (via either intravenous or intraorbital route) are considered after the failure of the conventional synthetic DMARDs [4]. Here in, we report a case of IOI treated with RTX monotherapy without the use of conventional synthetic therapy. 

## Case presentation

A 25-year-old woman with no past medical or surgical history presented to the ophthalmology clinic for the first time in September 2019 with unilateral swelling in the right orbit for more than two years. This swelling was associated with ptosis. She denied eye pain, diplopia, chemosis, joint pain, sinusitis, nasal or oral ulcer, nasal discharge, epistaxis, otitis media or hoarseness. None of her family members suffered from a similar condition or history of connective tissue diseases. She was single, a non-smoker, unemployed, and not on any regular or over-the-counter medications. 

**Figure 1:**
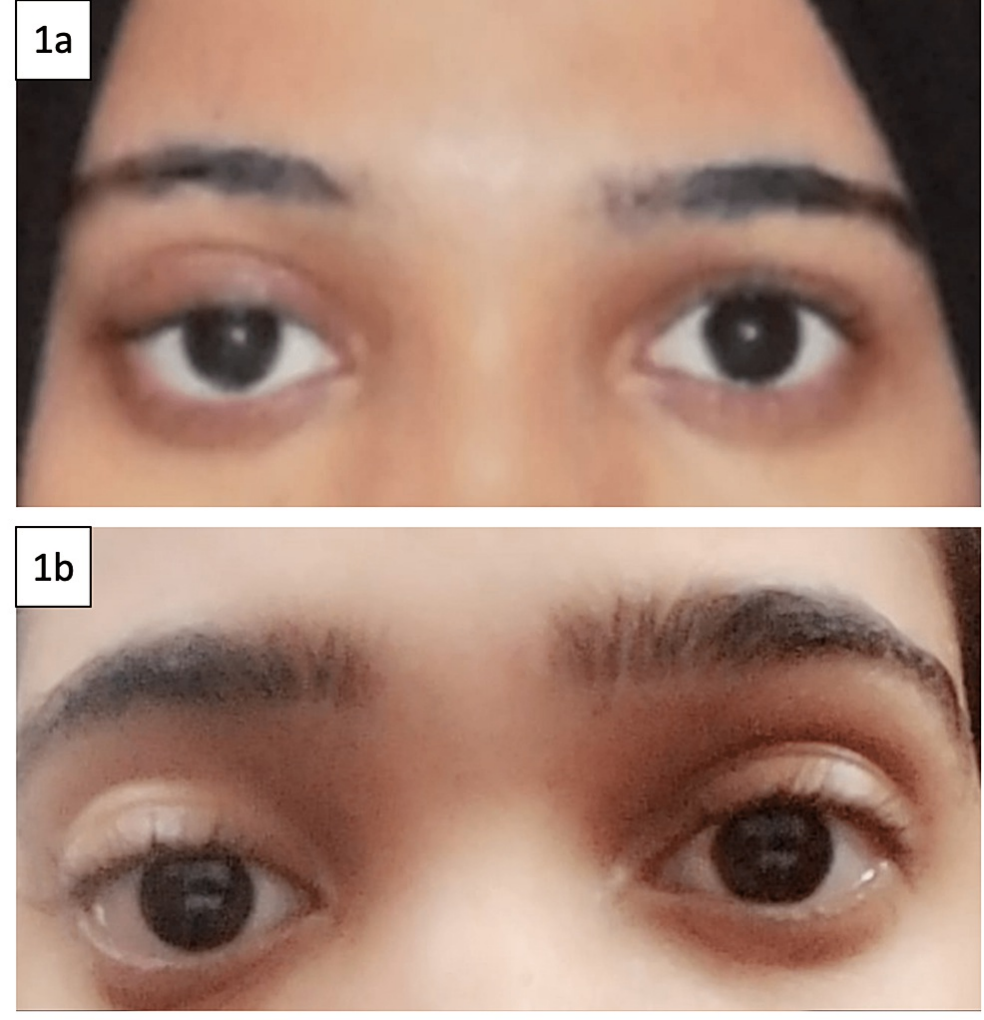
a: right orbital area swelling and ptosis of the right upper eyelid. b: clinical regression of the right orbital sweling and improvement of ptosis post three cycles of rituximab therapy.

Physical examination revealed proptosis of the right eye and ptosis, with swelling above the eyelid. Left eye examination was unremarkable (Figure 1a). She had no physical findings of an underlying systemic rheumatic disease. Laboratory investigations are shown in Table 1. 

**Table 1:** Baseline laboratory investigations for the patient.

Test	Result	Reference Range
Hemoglobin	132 g/L	120-160 g/L
White Blood Cell Count	7.9 10^9/L	3.5 – 10.5 x 10^9/L
Platelet Count	376 10^9/L	150 – 450 x 10^9/L
Aspartate Transaminase	14 U/L	8-48 U/L
Alanine Transaminase	9 U/L	7-55 U/L
Alkaline Phosphatase	49 U/L	45-115 U/L
Blood Urea Nitrogen	2.50 mmol/L	2.9 – 8.2 mmol/L
Creatinine	53 umol/L	50-110 umol/L
Creatinine kinase	54 U/L	20-60 U/L
Calcium	2.33 mmol/L	2.1-2.5 mmol/L
Immunoglobulin G subclasses	7.2 g/L	6-16 g/L

**Figure 2:**
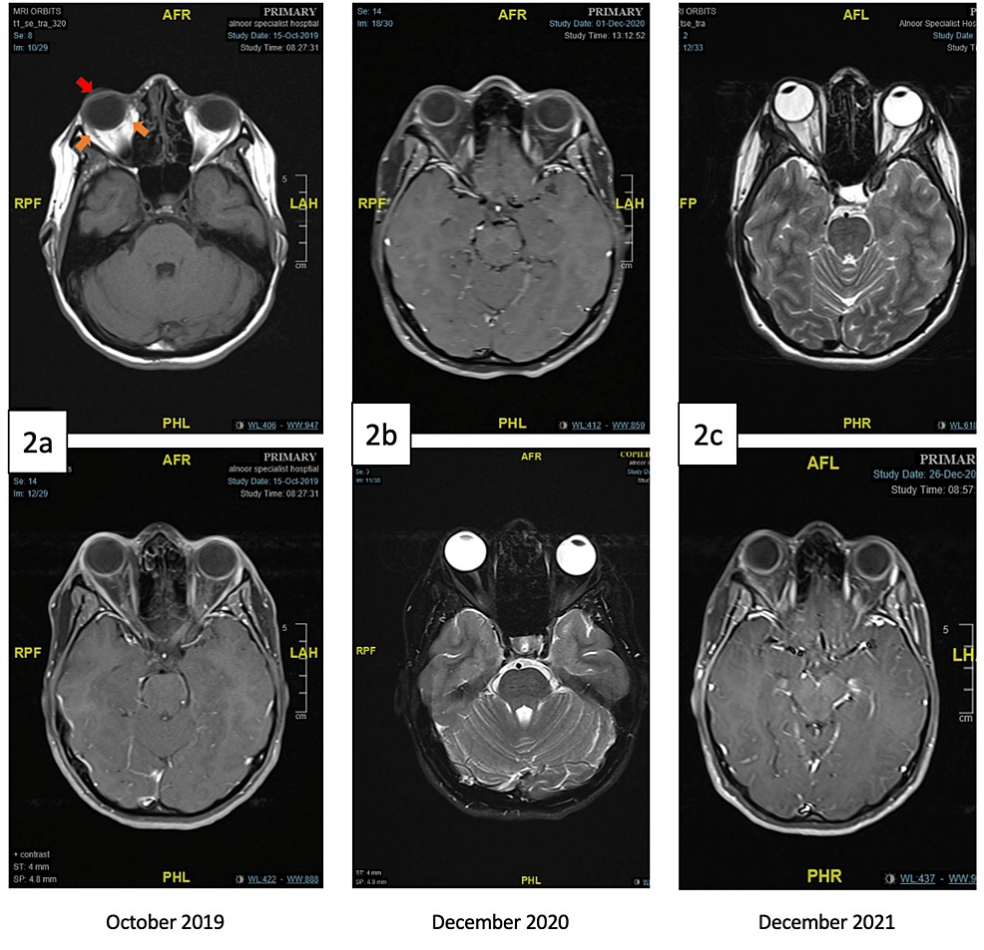
MRI of the orbit: a: (October 2019) significant proptosis of the right eye globe (red arrow) and swelling and enhancement of the extra-conal soft tissues (orange arrows), b: (December 2020) resolution of the proptosis and extra-conal findings post three cycles of rituximab therapy, c: (December 2021) sustained radiologic remission of the idiopathic orbital inflammation one-year post rituximab therapy.

**Figure 3:**
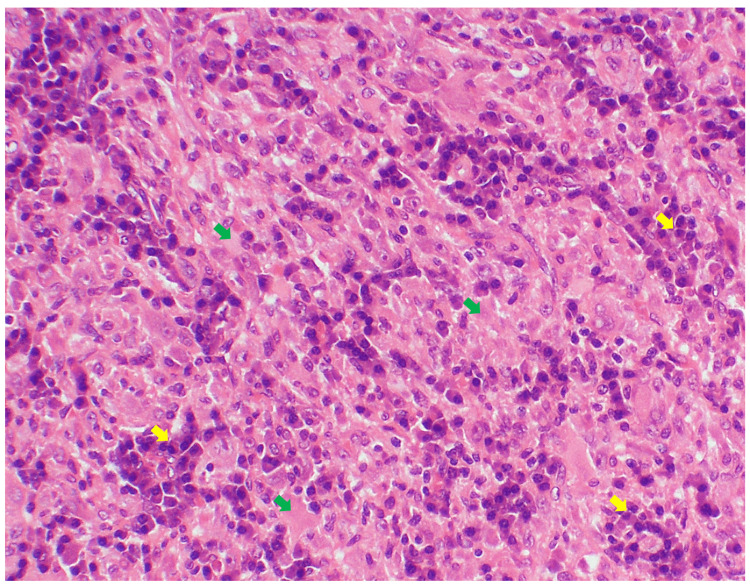
Right orbital mass histopathology: dense fibrous connective tissue and striated muscles (green arrows) and chronic inflammatory cell infiltrates (yellow arrows).

MRI of the orbit conducted in October 2019 revealed proptotic right eye globe with extra-conal enhancing soft tissue that is possibly involving the lacrimal gland (Figure 2a). For further assessment, biopsy of the right orbit was performed and showed fibrosis with CD20-positive inflammatory infiltrates, vascularized dense connective tissues and striated muscles (Figure 3).

Based on clinical, radiologic, and histopathologic assessments, the diagnosis of IOI was confirmed. Following a multidisciplinary discussion between ophthalmology, rheumatology and pathology teams and due to the abundance of the CD20 inflammatory infiltrates in the histopathology slides, RTX was started at a dose of 1000 mg intravenous (IV) infusions on days 1 and 15 (rheumatology protocol: a cycle of two sessions two weeks apart). This cycle was administered in December 2019, followed by two more cycles in June 2020 and December 2020, respectively.

Regular follow-up assessment revealed significant reduction in the right orbital swelling and improvement in the ptosis clinically (Figure 1b), resolution of the ptosis and extra-conal enhancement on repeat MRI after treatment (Figure 2b) and sustained clinical and radiologic remission one year post treatment (Figure 2c). 

## Discussion

IOI is a benign, inflammatory, non-infectious disorder that affects the orbit. It affects individuals of all ages and accounts for almost 5 to 7% of all orbital illnesses. It is the third most common type of orbital disease [5]. It is a rare disorder that has undergone minimal research internationally. This case is reported in Saudi Arabia and is nearly the most recent case report on IOI. 

The use of RTX in the management of IOI is supported by the pathophysiologic role of CD20 cells in the development of orbital fibrosis and presence of inflammatory infiltrates in the orbital structures. Review of the literature reports several cases of IOI treated successfully with RTX. A brief comparison between our case and the most recent case studies reported in the literature is presented in Table 2. This table shows the heterogeneity of the clinical presentation of IOI and the recorded variations in its management globally.

**Table 2:** Summary of clinical presentation and management of our case and the recent reported case of idiopathic orbital inflammation (IOI). RTX: rituximab

IOI cases	Clinical presentation	Management
Our case	28 years old woman Unilateral swelling in the right orbit for more than 10 years Proptosis of the right eye and ptosis MRI Orbit showed proptotic right eye globe with extra-conal enhancing soft tissue	3 cycles of RTX IV monotherapy
Case 1 [5]	67 years old man Chronic swelling of the left lower eyelid Normal visual acuity and ocular motility of the left eye Palpable mass at the lower eyelid	1.0–1.5 mg/kg of oral prednisone per day for 1 week and taper Intraorbital RTX at a dose of 10 mg once a week for the first month then a second complete cycle of 4 RTX IV injections
Case 2 [5]	54 years old man Right eye proptosis, diplopia in left lateral gaze and mild ptosis Visual acuity 0.2 logMaR in the right eye Palpable mass in thesuperotemporal orbital quadrant Inferomedial ocular globe displacement	1.0–1.5 mg/kg of oral prednisone per day for 2 weeks and taper Intraorbital RTX at a dose of 10 mg, followed by a complete cycle of RTX 4 months after the Intraorbital dose RTX
Case 3 [5]	41 years old woman Hashimoto’s thyroiditis on levothyroxine One-year history of pain in the superotemporal left orbit Visual acuity 0.2 logMaR in the left eye and 0.0 logMaR in the right eye Left ocular globe medially displaced by a palpable mass under the superotemporal orbital rim Upper eyelid erythema and edema, and ptosis and pain on left eye movements	1.0–1.5 mg/kg of prednisone per day for 1 week and taper Intraorbital RTX at a dose of 10 mg once a week for 4 weeks
Case 4 [6]	56 years old woman Hemochromatosis Painful, red, and swollen right eye Visual acuity 6/9 on the right, 6/6 on the left. Radiation of the pain to the right hemifacial and intracranial area Proptosis, excessive tearing, painful and restricted eye movements Intraocular pressure mildly elevated	Oral prednisolone for 12 months Azathioprine 100–150 mg and cyclosporin 100–300 mg) failed to produce meaningful results Methotrexate 10 mg weekly Targeted low-dose radiation therapy RTX 1000 mg intravenous infusion administered on day 1 and day 15, repeated every 6 months over a period of 3 years
Case 5 [7]	43 years old woman 17-Year history of seropositive rheumatoid arthritis 4-month history of diplopia Visual acuity 0.6 right eye and 0.0 left eye, best corrected on logMAR. Right-side proptosis, ptosis and diplopia on lateral gaze Restriction of movement in the right eye and reduction in vision Mild right-side proptosis and persistent choroidal folds affecting the right macula	Rheumatoid arthritis was very well controlled on adalimumab 3 pulses of IV methylprednisolone 250 mg Methotrexate 5 mg/week and folic acid 5 mg 3 days/week One cycle of RTX infusionsadministered 2 weeks apart

As noted in Table 2, the use of RTX for the management of corticosteroid-resistant orbital inflammation has widespread support in the current literature. RTX is used as a third-line agent in most patients with IOI who failed to respond to corticosteroids or other immunosuppressives [8,9]. However, it has been observed that the sooner the treatment with RTX the lesser the ophthalmic progression of the IOI. This was evident in cases 4 and 5 [6,7]. Both patients received RTX after the failure of conventional synthetic DMARDs by the time they had shown worsening of their symptoms in form of diplopia, restricted eye movement, and elevated intraocular pressure. In contrast, our patient and cases 1, 2 and 3 received RTX sooner in their disease course with or without prednisolone and hence did not experience disease progression [5].

## Conclusions

IOI is an uncommon but increasingly reported disease in the literature. Better understanding of the patho-etiology of IOI might help in the choice of targeted therapies for this disease. Early diagnosis and treatment are essential to prevent complications. In the absence of current guidelines for the management of IOI, RTX is an effective therapy for this disease, and it should be considered early in the disease course to prevent further ophthalmologic progression.
